# Trends in uptake of couples voluntary HIV counseling and testing (CVCT) in Lusaka and Southern Province, Zambia

**DOI:** 10.1186/1742-4690-9-S2-P208

**Published:** 2012-09-13

**Authors:** A Appiagyei, M Tufton, A Mwaanga, E Gudo, J Mubonde, W Kilembe, S Allen

**Affiliations:** 1 Zambia Emory HIV Research Project, Lusaka, Zambia; 2 Emory University School of Medicine, Atlanta, GA, USA

## Background

Couples HIV testing and counseling (CVCT) has been proven to decrease transmission of HIV in discordant couples by two-thirds. In order to achieve a snowball effect and establish CVCT as a social norm, the intervention must reach 15-20% of the target population. Considering demographics in Lusaka, Zambia, approximately 40,000 - 50,000 couples must be tested together within a few years to establish norms and set the stage for social diffusion.

## Methods

The authors investigated trends in couples seeking first time ZEHRP CVCT services in Lusaka and Southern Province government district clinics from January 2008 – December 2011. Trends in increases or decreases in uptake were compared to historical data such as changes in incentives for couples and CVCT promoters, as well as external events such as couples testing campaigns and government policies.

## Results

Over the study period, ZEHRP tested 37,859 couples. Declines in number of couples tested coincided with a discontinuation of performance based pay for District Clinic Promoters (DCPs) and a reduction in couples transport reimbursement. Though six new government clinics began offering ZEHRP CVCT in quarter four of 2008, no performance based pay or transport reimbursement were provided, and numbers were lowest at this time than at any point in the study period.

A sharp increase in CVCT uptake was seen in quarter one of 2010, which coincided with a mass media campaign for CVCT by another Zambian non-governmental organization, though uptake gradually declined once the campaign ended.

Number of couples seen in Southern Province remained stable throughout the study period, while most fluctuations were seen in Lusaka.

## Conclusion

Client and promoter incentives as well as mass media have been shown to influence uptake of CVCT. In urban areas where cost of living is high, CVCT programs must consider opportunity costs to encourage uptake.

